# How Do Object Shape, Semantic Cues, and Apparent Velocity Affect the Attribution of Intentionality to Figures With Different Types of Movements?

**DOI:** 10.3389/fpsyg.2020.00935

**Published:** 2020-05-15

**Authors:** Diego Morales-Bader, Ramón D. Castillo, Charlotte Olivares, Francisca Miño

**Affiliations:** Centro de Investigación en Ciencias Cognitivas, Facultad de Psicología, Universidad de Talca, Talca, Chile

**Keywords:** theory of mind, attribution of intentionality, agency, intentional stance, semantic priming, apparent speed

## Abstract

A series of experiments show that attribution of intentionality to figures depends on the interaction between the type of movement –Theory of Mind (ToM), Goal-Directed (GD), Random (R)– with the presence of human attributes, the way these figures are labeled, and their apparent velocity. In addition, the effect of these conditions or their interaction varies when the use of human nouns –present in the participant’s responses– is statistically controlled. In Experiment 1, one group of participants observed triangular figures (*n* = 46) and another observed humanized figures, called Stickman figures (*n* = 38). In ToM movements, participants attributed more intentionality to triangular figures than to Stickman figures. However, in R movements, the opposite trend was observed. In Experiment 2 (*n* = 42), triangular figures were presented as if they were people and compared to triangular figures presented in Experiment 1. Here, when the figures were labeled as people the attribution of intentionality only increased in R and GD movements, but not in ToM movements. Finally, in Experiment 3, Stickman figures (*n* = 45) move at a higher (unnatural) speed with higher frames per second (fps) than the Stickman figures of Experiment 1. This manipulation decreased the attribution of intentionality in R and GD movements but not in ToM movements. In general terms, it was found that the human attributes and labels promote the use of human nouns in participants’ responses, while a high apparent speed reduces their use. The use of human nouns was associated to intentionality scores significantly in R movements, but at a lesser extent in GD and ToM movements. We conclude that, although the type of movement is the most important cue in this sort of task, the tendency to attribute intentionality to figures is affected by the interaction between perceptual and semantic cues (figure shape, label, and apparent speed).

## Introduction

The use of moving figures to evaluate the attribution of intentionality derives from the classic study implemented by Heider and Simmel (1944). In this study, geometric figures in motion were presented in film format without sound. The people who watched these films described the animations with human character traits and mental states. Even though these animations were not designed to detect and encourage the attribution of mental states, based on that pioneering work; Abell et al. (2000) created a group of animations to study the attribution of intentionality; a task that is currently an experimental paradigm.

The task consists of 12 animations divided into three groups with different types of movements. Participants must observe, for a few seconds, videos in which a pair of geometric figures (Triangles) display a series of movements. Once each video is finished, they should describe what was observed. The descriptions usually include words that denote emotions, intentions, or mental states that the Triangles experienced while moving. The findings indicate that the use of words that refer to intentionality varies depending on the type of movement these figures display. In one type of movement, labeled as Random (R) movement, figures bounce in different directions without interacting with each other. In another type of movement, called Goal-Directed (GD), the figures act together, they move in a certain direction and show a synchronized interaction between them. Finally, in Theory of Mind (ToM) movements, the animations show the reaction of one of the figures to do something about a supposed state of mind of the other figure. The descriptions provided by participants have higher levels of intentionality as well as mental states when the figures display ToM movement than when they develop GD movement, and are more frequent with this type of movement than when they display R movement (Abell et al., 2000).

This type of task, where geometric figures are used, is based on the hypothesis that the characteristics of a moving object, such as its shape, is irrelevant to the emotional perception of the object (Michotte, 1950). The experiment implemented by Rimè et al. (1985) supported this hypothesis. They showed participants a series of films with geometric figures and different kinetic structures of GD movement. These participants found it more appropriate to describe the films in emotional words, both when geometric figures and human-shaped silhouettes were used. Therefore, Rimè et al. (1985) concluded that movement patterns were more important to the perception of emotional content than the appearance of the characters.

In line with this hypothesis, the evidence suggests that simple movement patterns involving changes in the speed and direction of objects, in the absence of some visible cause that can explain these changes, is enough for an object to be perceived as animated (Tremoulet and Feldman, 2000; van Buren et al., 2015). Surian and Caldi (2010) concluded that 10-month-old children can detect autonomous agents based on movement even before they can categorize what they see. Therefore, movement by itself seems to be more important than the shape of objects to perceive them as animated and attribute emotionality to them.

However, in other contexts, form or appearance does relate to the perception of intentionality. In research with anthropomorphized robots, the likelihood of building a model of someone else’s mind increases with its perceived human-likeness (Krach et al., 2008). People empathize more strongly with human-like robots and less with functional robots (Riek et al., 2008). When the human-likeness increases, people’s Intentional Stance (IS) toward a robot could be very similar to their intentional stance toward a human (Thellman et al., 2017). The intentional stance is a concept linked to the ToM. According to Dennett (1971), there are at least three strategies or stances to explain and predict other entities’ behaviors (living or non-living things) that can be based on design stance, physical stance, or intentional stance. Intentional stance relies on the ascription of mental states to a system in order to explain and predict its behavior. These mentalist ascriptions might be accurate or erroneous; and in that regard, the intentional stance is not subject to the same requirements as the ToM to be valid. The person may incorrectly detect a character’s false belief in ToM tasks and still attribute intentionality to the behavior (Marchesi et al., 2019). Thus, while ToM requires that the person correctly identify what is the other’s mental state (beliefs, desires, thoughts, feelings, among others), for the intentional stance it is only necessary that the person attributes the behavior to a mental state, whatever it may be. Even when these studies show the importance of anthropomorphic features in attributed intentionality toward humanoid robots (or intentional stance and emotional reactions); there is no information about what could happen when these human-like devices deploy movements that are considered the most important cues for attribution of intentionality, as it is the case of the animations of Abell et al. (2000).

Another variable that could affect the attribution of intentionality, and that is not usually considered in studies with the triangles task (Abell et al., 2000), is the label of the figures or the instructions for the task. For example, the labels that researchers put on their video stimuli may favor the attribution of intentionality. Oatley and Yuill (1985), using a version of the moving geometric figures task, found that people used more descriptions of mental states when animations had titles like “Jealous Lover.” The meanings associated with a label in a video stimulus affected the attribution of emotions to the objects that appeared in that video. In an experiment conducted by Wiese et al. (2013), in which the humans-robots interaction was analyzed, participants looked at a human face and a robot face. When participants were led to believe they were observing an intentional behavior, the gaze times were significantly longer compared to when they were led to believe that the behavior was pre-programmed. The effect of adopting the intentional stance occurred regardless of whether a human face or a robot face was presented. Özdem et al. (2017) led participants to believe that the eye movements of a robot face were controlled by a human rather than being pre-programmed. The belief that the eye movements are controlled by a human seems to attract more attention and appear to be more socially relevant and informative than the belief that the movements are pre-programmed. Therefore, the intentionality attribution increases when participants are provided with information with certain semantic cues in instructions.

In our review, we have detected other factors that influence the attribution of intentionality or the intentional stance. However, an analysis of how these factors or other sources of variability, derived from how the task developed by Abell et al. (2000) has been administered to the participants, has not been performed. The variations detected range from the way the figures are described to providing details about the three types of movements that these figures display. For example, Castelli et al. (2000) told participants that the Triangles acted as characters making different movements. In addition, they described each of the three types of movements of the Triangles. Salter et al. (2008) explained to participants that the Triangles were acting as characters and that they could be doing something together or something more complicated like thinking about each other’s feelings. However, they did not mention the types of movements in the task (ToM, GD, and R). Zwickel et al. (2011) asked participants to make verbal descriptions of the videos, but, they did not provide any content related to the type of movement of the animations. Other studies have used a forced-choice paradigm; where participants were instructed to watch the videos and then had to choose among three types of responses: “no interaction” (R movement), “physical interaction” (GD movement), and “mental interaction” (ToM movement) (White et al., 2011; Brewer et al., 2018).

In our opinion, the problem of providing information about the types of animations, such as exemplifying with mental or emotional states –as it happens in certain training videos– or switching between a forced-choice paradigm to an open-ended response one, is that these manipulations could inhibit or stimulate the attribution of intentionality to certain types of movements.

Providing information that promotes such attribution could potentially affect the ability to detect the effect of relevant variables such as those we analyzed in this study. We found that this problem was partially addressed by Klein et al. (2009). In their study of fixation times in each type of animation, they also explored whether previous expectations could cause bias in participants. The first 10 participants received only general instructions, and the next 10 received additional information on the three types of movements in practice trials. The last 11 participants also received information on what type of animation they would see in each video before the animation was shown, but this information was only shown in the practice trials, not during the entire experiment. Klein et al. (2009) found no significant differences in the instructions given to participants regarding the intentionality of verbal descriptions and fixation times, without providing greater background to assess the significance of these results.

Based on this preliminary background information, it is possible to state that providing details that favor the attribution of intentionality does not affect the observed trend among types of movements. However, when studies that have used the same scales to determine intentionality (from 0 to 5 points) are compared; it is possible to observe that the values for figures displaying ToM movement range from 2.73 to 3.80; for figures displaying GD movement the values range from 2.21 to 2.64; whereas for figures displaying R movement the values of intentionality range between 0.12 and 0.47 (Castelli et al., 2000; Salter et al., 2008; Klein et al., 2009; White et al., 2011; Zwickel et al., 2011). Studies tend to replicate that people attribute more intentionality to figures with ToM movement than to figures with GD movement, and more intentionality to this type of movement (GD) than to R movements.

Hence, the gradient of intentionality that occurs between types of movement is fairly stable in several studies. However, variation in scores is a factor that could be caused by variability in instructions, how the participant is required to respond, or other subtle aspects. Instructions that use certain words or grammatical structures may lead participants to use those same words or grammatical structures, a phenomenon known as syntactic priming (Scarborough et al., 1977; Sereno and Rayner, 1992). If people hear the researcher talk to them about characters who think, feel, cheat or play, they are likely to use the same words, and in the same grammatical forms, to describe what they see. One way to control this effect would be to use standardized instructions that do not provide information that might induce the attribution of intentionality by factors other than the movements of the figures.

A less studied factor that could have an important effect when it comes to explaining variations in attributions of intentionality is the frequency of frames or images per second (fps) at which animations are displayed. Morewedge et al. (2007) observed that participants perceived that targets (animals, robots, and animations) are more likely to appear to possess mental states when they move at speeds similar to natural human movement, compared to targets that perform actions at faster or slower speeds. They varied the apparent speed of objects, as well as the number of fps of non-human animations (slow movies were presented at 1 fps, medium movies at 6.60–11.60 fps, and fast clips at 16.60–50.00 fps). In a review, Chen and Thropp (2007) compiled the effects of fps on psychomotor performance, perceptual performance, behavior and subjective perception tasks. They found that individuals appear to be able to gather information about the content of videos that are viewed at very low fps (5 fps). This could benefit the assimilation of information because each frame stays longer on the screen compared to videos that are presented at higher rates. Thus, viewers would have more time to observe and process each frame. Our analysis of the figures of Abell et al. (2000) determined that they have an average of 10 fps, five more than those reported by Chen and Thropp (2007) and within the intermediate category in the study by Morewedge et al. (2007). Under this precedent, it would be possible to state that presenting videos with more fps could have a negative effect on the attribution of intentionality.

Therefore, empirical evidence indicates that humans attribute intentionality to simple images, even at an early age. This evidence further indicates that the shape of figures is not relevant in terms of attribution of intentionality when an object moves in a GD manner. Thus, a figure with human characteristics would have the same effect as an inanimate object in a GD movement. However, when three-dimensional objects are used and these objects mimic human features, like a humanoid robot, people are more prone to attribute intentionality to them than to less human-like objects. On the other hand, it has been demonstrated that the type of movement shown by the figures is a variable that systematically reproduces the same result. Figures showing R movements get low scores of intentionality. Intentionality scores increase progressively with GD movements and most notably in figures that show ToM movements. Furthermore, when videos are labeled with words alluding to an emotional state, the descriptions of those videos include more mental states than when they are not labeled. The results also show variability in responses that could be attributed mainly to the instructions that differ between the studies analyzed. Another explanation could be that the apparent speed of the animations may decrease the attribution of intentionality when it is too slow or too fast compared to what is expected or what is closer to natural human movement.

We conducted three experiments to evaluate the influence of the figure’s shape, the label used to nominate the figure, the apparent speed of the figure’s movement, and the interaction of these attributes with the type of movement. There is a lack of information about the relationship of these variables with the attribution of intentionality. These variables may be present in studies using the Frith-Happé triangles task, or similar tasks where people have to attribute intentionality to moving figures (e.g., Ramsey and Hamilton, 2010; Surian and Geraci, 2012; van Buren et al., 2015). Theoretically, it has been demonstrated that these variables affect the attribution of intentionality, regardless of the movement’s effect. Methodologically, we found significant variability in the way the Frith-Happé triangles task is applied. Studying these variables may help control unwanted effects on attribution of intentionality.

In terms of the figure’s shape, we hypothesized that the human-like figures (Stickman) would be attributed more intentionality than the abstract figures (Triangles). We expected that this core effect will be primarily sustained by differences in ToM and R movements. However, no differences were expected in GD movements, since attribution of intentionality, animated movement perception, or goal attribution, have been fairly stable in previous research, whether or not the stimulus was morphologically human or non-human (Michotte, 1950; Rimè et al., 1985; Johnson et al., 2001; Shimizu and Johnson, 2004; Shultz et al., 2011). In terms of the figure’s label, we expect that those referred to as a person (Mr. X and Mr. Y) would generate more intentionality than abstract figures labeled as objects (Figures). Regarding the apparent speed of movement, we hypothesized that figures moving at low apparent speed (10 fps) would be attributed more intentionality than figures moving at high apparent speed (23 fps). Finally, we conjectured that the type of motion interacts with these three attributes (shape, label, and apparent speed); even when the gradient of intentionality observed between the types of movements (ToM > GD > R) is quite stable.

In addition, we measured the use of human nouns to assess the relationship of these nouns with the attribution of intentionality. Considering that instructions that include examples and explanations might be factors that facilitate the use of human nouns and at the same time promote the use of the IS, we simplified the instructions to avoid giving additional information. Our hypothesis about human nouns was that figures and label shape would facilitate the use of these nouns, which would also be reflected in a greater attribution of intentionality. However, we expected that by controlling this effect the differences observed by the shape of the figures and labels would be reduced to some extent.

## Experiment 1: the Effect of Anthropomorphized Figures on Attribution of Intentionality

### Method

Participants were assigned to two conditions with different types of figures (Triangles or Stickman figures). These figures appeared in three types of movements (ToM, GD, and R). Each type of movement contained four different videos. A total of 12 videos were watched. After each video, participants had to write a description of what they had seen on the computer. Responses were categorized according to degrees of intentionality (Abell et al., 2000; Castelli et al., 2000; Salter et al., 2008; Zwickel et al., 2011). In addition, the use of human nouns was counted for each video.

Sample size estimation was made using the G-Power software (Faul et al., 2007). For each experimental condition, the minimum sample size was estimated using group comparison repeated measures ANOVA, where the between-subject factor (two groups) interacts with a within-subject factor (three types of movement). Thus, based on an effect size = 0.51; an error α = 0.05; and a power (1−β) = 0.95, the minimum sample size was set at 34 participants. Hence, we selected samples with at least 34 participants in each condition for all experiments.

### Participants

The sample consisted of 84 university students: 51 women and 33 men between the ages of 18 and 29 (*M* = 20.4 years, SD = 2.4 years). Participants were randomly assigned to the Triangles (*n* = 46) and the Stickman (*n* = 38) groups. All participants read and signed the informed consent form before the experiment, approved by the Ethics Committee of the University of Talca (FONDECYT 1161533).

### Stimuli and Apparatus

Frith-Happé animations were used (Castelli et al., 2000, 2002), consisting of twelve animations with a duration of 34–45 s each, and three practice and familiarization animations. These animations include two triangles, one large and red and the other small and blue, that perform different actions and movements on a plane with a white background. There are three groups of movements with four videos each: ToM, GD, and R. The ToM videos shows the two Triangles performing movements that give the impression of seducing, cheating, making jokes, and being surprised. The GD videos show the two Triangles dancing together, chasing each other, fighting, and guiding or leading. Finally, the R movement videos show the two Triangles bouncing on the walls or simply moving around the plane as cause and effect reactions.

**FIGURE 1:**
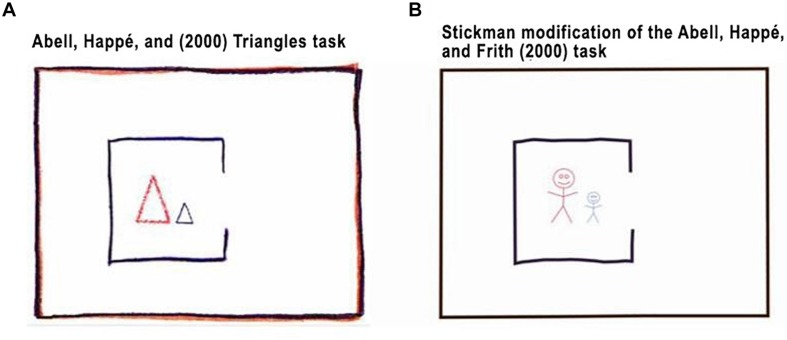
Comparison of the figures used in our experiments. Panel **(A)** shows the figures used in the original Abell et al. (2000) triangles task. Panel **(B)** shows our modified figures.

To produce the condition of the Stickman figures, the original task was modified using Adobe After Effects CS6 video editing software, changing the Triangles to humanized figures (see Figure 1). The paths, turns, size, and shape changes of the Triangle movements of the original task were replicated.

The subjects had to describe what was happening in each video. These descriptions were scored according to degrees of intentionality based on criteria defined by Abell et al. (2000). Intentionality was scored between 0 and 5, based on the verbs in the participants’ responses. At the bottom of the scale, there is no recognition of the other figure, nor deliberate actions or mental states (score = 0). The actions are involuntary, and there is no interaction between them, the figures move randomly. One step higher on the scale (score = 1), a figure acts with a purpose or goal, without interaction with the other figure. The next level (score = 2) is when a figure acts with a purpose in conjunction with the other figure. The actions of the figures are parallel in time. At the next level (score = 3), the figure not only interacts with another figure but also acts in response to the action of the other figure. The actions of both figures are sequential in time. Finally, the two scores at the top of the scale consist of describing the mental states of the figures. A score of “4” is when the figure acts in response to a mental state or reveals that it has a mental state. The highest score on the scale (score = 5) is when one figure acts with the aim of affecting or manipulating the mental states of the other.

The experiment was conducted using the open-source experiment builder “*OpenSesame*” (Mathôt et al., 2012). The instructions were shown to participants, indicating that they would see a series of animations with two figures as protagonists and that after each animation they should simply describe what was happening in each video. Participants were not told that there were three types of movements, but they were presented with one of each condition as a practice and familiarization trial, without providing them with further details. After the familiarization trials, participants were instructed to watch twelve animations similar to the prior ones and type in the text box shown by the program after each animation their answer to the following question: *What was happening in the video?*

The presentation of the videos was pseudo-random, with the criterion that no more than two animations of the same condition were repeated sequentially. Randomization with these criteria was performed using the “*Mix*” program (van Casteren and Davis, 2006), generating 18 different lists of the order of appearance of the animations.

### Inter-Rater Reliability for Answer Coding Criteria

A total of 10 undergraduate psychology students were trained as raters to categorize verbal responses. In a 3-h workshop, they learned to use the criteria to categorize intentionality to discriminate between the different levels of each variable. Thirty-six responses were randomly selected for the test, covering all score levels. Eighteen responses were used in the training. Once the raters assessed the responses in terms of their degree of intentionality, the Inter-Rater Agreement (IRA) coefficient by Cohen et al. (1992) was estimated. The minimum IRA to be considered suitable with ten judges is 0.62 and that value is only possible if 9 out of 10 judges agree. The IRA average for intentionality in this experiment was 0.68, with 82% of agreement.

In addition, Kendall’s W coefficient of concordance was estimated for three raters who analyzed two series of responses that had previously been categorized according to their degree of intentionality. Two evaluations of six written responses were developed. They had previously been selected as being representative of each degree of intentionality (from 0 to 5). The values were significantly different from the expected random level of agreement, varying between W = 0.65; $\chi_{\left(2\right)}^{2}$ = 11.19, *p* = 0.048 for the first series of written responses, and W = 0.95, $\chi_{\left(2\right)}^{2}$ = 14.24; *p* = 0.014 for the second series. Considering the values, it can be concluded that the criteria used to describe degrees of intentionality were clear enough for a group of raters, trained in the use of those criteria, to consistently classify responses, exceeding the minimum agreement value. In that regard, there was a high level of inter-rater agreement in the correct application of the criteria of intentionality.

### Results

A 3-by-2 mixed ANOVA was implemented. The intra-subject factor corresponded to the type of movement in the animation (ToM, GD, and R), and the inter-subject factor corresponded to the type of figure (Triangles and Stickman figures). For this and all other experiments, Huynh-Feldt’s correction was applied whenever Mauchly’s test indicated that the assumption of sphericity was not met. Once an interaction effect was detected, the simple effects were evaluated with a *post hoc* analysis using the Bonferroni correction.

**FIGURE 2:**
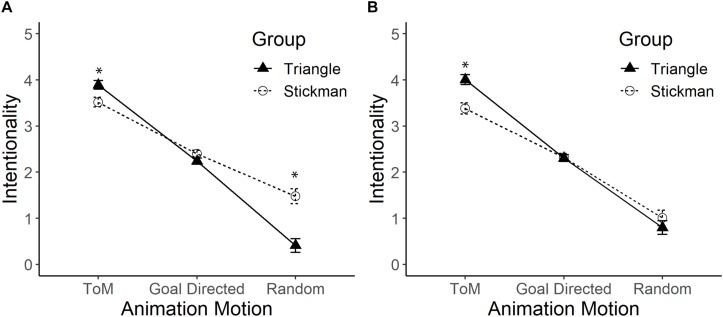
Mean and standard error of the Triangles group and the Stickman figures group on the intentionality scale in the Theory of Mind (ToM), Goal-Directed (GD), and Random (R) movements. Panel **(A)** shows the comparison between the Triangles and the Stickman figures. Panel **(B)** shows the comparison when statistically controlling the effect of the use of human nouns. The asterisk (*) indicates significant differences. Pearson’s Correlation between intentionality in each type of movement with the score for the use of human nouns (from 1 to 12), was positive in R movements (*r* = 0.64, *p* < 0.01), and GD movements (*r* = 0.26, *p* = 0.01). However, in ToM movements (*r* = –0.02, *p* = 0.85) there was no correlation between intentionality scores and the use of human nouns.

The results showed an effect of interaction between the type of figure and the type of movement, *F*(2,164) = 32.71, *p* < 0.01, $\eta_{\text{p}}^{2}$ = 0.29. The *post hoc* analysis (see Figure 2, Panel A) found that participants who watched Triangles (*M* = 3.89) attributed greater intentionality than those who watched Stickman figures (*M* = 3.52) when the figures displayed ToM movement, *F*(1,82) = 6.90, *p* = 0.1, $\eta_{\text{p}}^{2}$ = 0.08. The opposite occurred when the figures displayed R movement, since participants who watched Triangles (*M* = 0.41) attributed less intentionality than those who watched Stickman figures (*M* = 1.48), *F*(1,82) = 24.25, *p* < 0.01, $\eta_{\text{p}}^{2}$ = 0.28. However, when the figures displayed GD movements, there were no differences between those who watched Stickman figures (*M* = 2.40) and those who watched Triangles (*M* = 2.24), *F*(1,82) = 2.79, *p* = 0.10. There was a gradient of intentionality in the movement type scores, *F*(2,162) = 468.71, *p* < 0.01, $\eta_{\text{p}}^{2}$ = 0.85, where the attribution of intentionality was greater with ToM movement (*M* = 3.71) than with GD movement (*M* = 2.32), *p* < 0.01 and the intentionality for this type of movement was higher compared to that of R movement (*M* = 95), *p* < 0.01. Finally, differences between type of figure were observed, where the Stickman figures (*M* = 2.47) had more attribution of intentionality than the Triangles (*M* = 2.18), *F*(1,82) = 5.99, *p* = 0.02, $\eta_{\text{p}}^{2}$ = 0.06.

From the responses written in each of the 12 animations, it was possible to detect that 8.7% (4 out of 46) of those who observed Triangles used human nouns; while 76.3% (29 out of 38) of those who observed Stickman figures used human nouns. These differences are statistically significant, $\eta_{\text{p}}^{2}$ = 39.89, *p* < 0.01. However, in both conditions, participants who watched Triangles [*t*_(45)_ = −9.83, *p* < 0.01] and Stickman figures [*t*_(37)_ = 3.77, *p* < 0.01] part of the responses significantly differed from what was randomly expected. With these results, it was concluded that experimental manipulation promoted the use of human nouns with Stickman figures and disincentivized their use with the Triangles. Taking this information into account, an ANCOVA was conducted, statistically controlling the effect of the use of nouns.

The use of nouns turned out to be statistically significant on its own, *F*(1,81) = 18.86, *p* < 0.01, $\eta_{\text{p}}^{2}$ = 0.19 and, at the same time, it interacted with the type of movement, *F*(2,162) = 7.55, *p* < 0.01, $\eta_{\text{p}}^{2}$ = 0.09. Statistically controlling the effect of this variable, an interaction effect between the type of figure and the type of movement was again detected, *F*(2,162) = 8.65, p < 0.01, $\eta_{\text{p}}^{2}$ = 0.10 (see Figure 2, Panel B). Participants who watched Triangles (*M* = 4.05) attributed greater intentionality than those who watched Stickman figures (*M* = 3.33) when the figures displayed ToM movement, *F*(1,81) = 14.61, *p* = 0.001, $\eta_{\text{p}}^{2}$ = 0.15. There were no differences between Stickman figures and Triangles when the figures displayed GD movement, *F*(1,81) = 0.097, *p* = 0.76, and R movement, *F*(1,81) = 0.88, *p* = 0.35. The intentionality gradient in the type of movement variable was consistently maintained, *F*(2,162) = 257.34, *p* < 0.01, $\eta_{\text{p}}^{2}$ = 0.76, where the attribution of intentionality was greater with ToM movement (*M* = 3.69) than with GD movement (*M* = 2.31), *p* < 0.01 and the intentionality attribution for this type of movement was higher compared to that of R movement (*M* = 91), *p* < 0.01. However, no differences were found by type of figure, so people who watched the Stickman figures (*M* = 2.22) attributed similar intentionality compared to participants who watched the Triangles (*M* = 2.39), *F*(1,81) = 1.21, *p* = 0.27.

### Discussion

In this experiment, we contrasted the effect of the figure’s shape on the attribution of intentionality. It was assumed that people would be insensitive to the type of figure in GD movements (Michotte, 1950; Rimè et al., 1985). However, we expected that the subjects who watched the Stickman figures would assign higher intentionality scores in the ToM and R movements. We found higher scores of intentionality in R movements in the group that watched the Stickman figures, but, contrary to our hypothesis, this group attributed lower intentionality scores in ToM movements compared to the group that watched the Triangles. Additionally, it was also found that people used more human nouns when describing the Stickman figures than when describing the Triangles, even though they had both been treated as figures. When the use of nouns was statistically controlled, the Stickman figures were still attributed less intentionality than the Triangles in ToM movement animations, whereas the differences in the R movement disappeared. This suggests that the use of human nouns would lead to greater attribution of intentionality only in R movements. The most important effect of this experiment was the interaction between the type of movement and figure. This interaction varied slightly when the effect of human nouns was controlled.

The results reveal that the type of figure does matter when attributing intentionality to objects, but that this importance is only present when the figure develops ToM and R movements. The studies we reviewed suggested that there would be no significant differences, but they only evaluated this hypothesis in figures with GD movement (Michotte, 1950; Rimè et al., 1985). In our case, we included two additional movements, ToM and R, making the experimental manipulation more prone to interaction. In addition, this interaction effect was maintained even when the use of human nouns was controlled, which is why it does not depend on the ability of certain words to induce descriptions of intentionality, as it does in R movements.

Contrary to what we expected, the Triangles were attributed more intentionality than the Stickman figures displaying ToM movements. This finding may indicate that the figure may have distracted the participants from grasping the main cue for the attribution of intentionality, which is the type of movement. We believe that this result has more to do with the design of the figure or the discrepancy of the figure with the movements (e.g., smiling faces that do not change their expression with the movements) than with the human anthropomorphism of the figure.

Experiment 2 evaluated the effect of figure labeling on intentionality attribution. We expected higher intentionality scores in the group in which the figures were treated as people. Considering the results of Experiment 1, we expected the differences in intentionality scores to be substantial in the R movements due to greater use of human nouns.

## Experiment 2: the Effect of Figure Labeling on Attribution of Intentionality

Experiment 2 aimed to assess the effect of labeling abstract figures as people could have on the attribution of intentionality. Experiment 1 showed that anthropomorphized figures in human-like form interacted with the type of movement, but only when the type of movement was ToM and R. In this experiment, as in the first one, it was expected to cause a similar effect by manipulating the way the figure was referred to. In this case, the Triangles were referred to as figures in one condition, and as people in the other condition. We hypothesized that with standardized instructions, in figures referred to as people, the use of human nouns would be stimulated. But by statistically controlling the use of these nouns, differences in terms of intentionality would be reduced. And if there were interaction effects, there would be differences between the ways they were referred to, but only in certain types of movements.

### Participants and Procedure

A total of 42 participants (28 men, 14 women) with an average age of 20.8 years (SD = 2.1), watched the original Frith-Happé animations, but the description indicated that the red triangle was referred to as “Mr. X” and the blue triangle was “Mr. Y.” As a comparison group, the sample group from Experiment 1 (*n* = 46) that watched Triangles referred to as Figure X and Figure Y, respectively, were used.

### Results

As in Experiment 1, a 3-by-2 mixed ANOVA was conducted. The intra-subject factor corresponded to the type of movement (ToM, GD, and R), while the inter-subject factor corresponded to how Triangles were labeled (as a person, Mr. X and Mr. Y versus as figures, Figure X and Figure Y).

**FIGURE 3:**
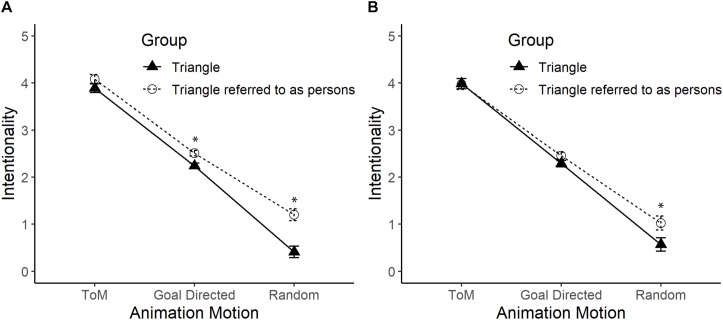
Mean and standard error of the Triangles group and the Triangles referred to as people group on the intentionality scale in the ToM, GD, and R movements. Panel **(A)** shows the comparison between the Triangles and the Triangles referred to as people. Panel **(B)** shows the comparison by statistically controlling the effect of the use of human nouns. The asterisk (*) indicates significant differences. Pearson’s Correlation between intentionality in each type of movement with the score for the use of human nouns (from 1 to 12) was higher in R movements (*r* = 0.44, *p* < 0.01), and lower in GD (*r* = 0.33, *p* = 0.001), and ToM movements (*r* = 0.23, *p* = 0.03).

An effect of interaction between the label of the figure and the type of movement was detected, *F*(2,172) = 8.82, *p* < 0.01, $\eta_{\text{p}}^{2}$ = 0.09 (see Figure 3, Panel A). The *post hoc* analysis found that participants attributed more intentionality to the Triangles referred to as people (*M* = 2.51) than to the Triangles referred to as figures (*M* = 2.24), when the movements were GD, *F*(1,86) = 11.14, *p* < 0.01, $\eta_{\text{p}}^{2}$ = 0.12. The same trend was observed when R movements were displayed, because the Triangles referred to as humans (*M* = 1.20) were attributed higher levels of intentionality than the Triangles referred to as figures (*M* = 0.41), *F*(1,86) = 20.36, *p* < 0.01, $\eta_{\text{p}}^{2}$ = 0.19. However, with ToM movements, both Triangles referred to as people (*M* = 4.08) and Triangles referred to as figures (*M* = 3.89) were attributed similar levels of intentionality, *F*(1,86) = 2.07, *p* = 0.15. In addition, a main effect of the type of movement was detected, *F*(2,172) = 839.44, *p* < 0.01, $\eta_{\text{p}}^{2}$ = 0.91, where the attribution of intentionality was greater with ToM movement (*M* = 3.99) than with GD movement (*M* = 2.38), *p* < 0.01; and with this movement the attribution of intentionality was higher compared to that of the R movement (*M* = 81), *p* < 0.01. There were also differences depending on the way the Triangles were labeled, so that the Triangles referred to as people (*M* = 2.60) had higher levels of intentionality than the Triangles referred to as figures (*M* = 2.18), *F*(1,86) = 16.86, *p* < 0.01, $\eta_{\text{p}}^{2}$ = 0.16.

From the participant’s responses, it was possible to establish that 33 out of 42 (78.6%) of those who observed Triangles referred to as people used human nouns in at least one of the 12 videos. This value differed from 4 out of 46 (8.7%) who used human nouns when they observed Triangles referred to as figures, $\chi_{\left(1\right)}^{2}$ = 43.99, *p* < 0.01. As established in Experiment 1, participants who watched Triangles referred to as figures tended not to use human nouns [*t*_(45)_ = −9.83, *p* < 0.01], while in this experiment, subjects who watched Triangles referred to as people tended to use human nouns above what was randomly expected [*t*_(41)_ = 4.46, *p* < 0.01]. Thus, as in Experiment 1, the use of nouns was incorporated into the model as a covariate and an ANCOVA was conducted, statistically controlling its effect.

The use of nouns was statistically significant, *F*(1,85) = 5.28, *p* = 0.024, $\eta_{\text{p}}^{2}$ = 0.06, but did not interact with the type of movement, *F*(2,170) = 1.29, *p* = 0.28. Statistically controlling this variable, the previously observed interaction between the label of the figure and the type of movement tended to disappear, *F*(2,170) = 2.47, *p* = 0.09, even though in R movement there was more attribution of intentionality with the Triangles referred to as people than with the Triangles referred to as figures, *p* < 0.01 (see Figure 3, Panel B).

The gradient of intentionality was maintained by type of movement, *F*(2,170) = 414.29, *p* < 0.01, $\eta_{\text{p}}^{2}$ = 0.83, with attribution of intentionality being greater in ToM movement (*M* = 3.98) than in GD movement (*M* = 2.37), *p* < 0.01; and the intentionality attributed to this type of movement was higher compared to that in R movement (*M* = 0.80), *p* < 0.01. Finally, according to how the Triangles were labeled, there were no differences, so that the Triangles referred to as people (*M* = 2.49) had similar levels of intentionality compared to the Triangles referred to as figures (*M* = 2.28), *F*(1,85) = 2.24, *p* = 0.14.

### Discussion

As in Experiment 1, the differences according to the type of movement were very stable since the same gradient of intentionality was observed (ToM > GD > R). As expected, we found that participants who watched the figures referred to as people generally attributed greater intentionality, but this occurred only in the GD and R movements, not in the ToM movements. This group of participants also used more human nouns in their responses. By controlling this effect, the interaction between the label of the figures and the type of movement disappeared. We found that, in this case, only the differences in R movements were maintained.

These results suggest that the most important effect of the human label given to the figures was the greater use of human nouns in the responses of participants, which increased the attribution of intentionality in GD and R movements, but not in the ToM movements. These results also indicate that just by showing a human label, regardless of whether participants use human nouns, people will attribute greater intentionality in R movements.

It was hypothesized that using words that referred to figures as people, would act as semantic priming, inducing participants to use similar words or grammatical structures (Scarborough et al., 1977; Sereno and Rayner, 1992). That is, if participants read that the instructions suggested they were in the presence of people, this would serve as priming to describe the figures as entities that thought, felt, or possessed mental states, and to use the same grammatical forms to describe the movements of the figures. It was also expected that this priming would be sensitive to the control of the effect of human nouns and that, once this effect was eliminated, the differences in intentionality between the Triangles referred to as people and as figures would disappear. However, an interaction was also expected, which in this experiment was very subtle, but suggests that humans are sensitive to the interaction between the movement of the figure and the way it is labeled.

## Experiment 3: the Effect of Apparent Velocity (fps) on the Attribution of Intentionality

The objective of Experiment 3 was to evaluate how the fps of the animations could have an important effect in explaining the variations in the attributions of intentionality. Morewedge et al. (2007) found that people attribute less intentionality when animations have high fps (higher apparent speed). Our analysis of Abell et al. (2000) figures determined that they had an average of 10 fps, which is within the intermediate range of the Morewedge et al. (2007) study. In this context, it was hypothesized that showing videos with more fps could have a negative effect on the attribution of intentionality.

### Participants and Procedure

A total of 45 subjects (11 men and 34 women), with an average age of 19.6 years (SD = 1.7), watched Stickman figures with high fps (26 fps on average). As in Experiment 1, the instruction asked them to make a description of what they had seen. As a comparison, the sample group from Experiment 1 (*n* = 38) which watched Stickman figures with approximately 10 fps, was used.

### Results

In this experiment, the intra-subject factor corresponded to the type of movement of the animations (ToM, GD, and R), while the inter-subject factor corresponded to the fps of each figure (original Stickman figures with 10 fps and Stickman figures with 26 fps).

**FIGURE 4:**
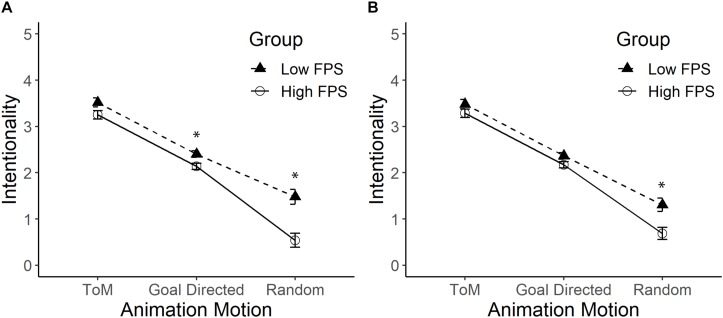
Mean and standard error of intentionality for Stickman figures displayed at low fps rate and high fps rate with ToM, GD, and R movements. Panel **(A)** shows the comparison between the Stickman figures displayed at a low fps rate and the Stickman figures displayed at a high fps rate. Panel **(B)** shows the comparison by statistically controlling the effect of the use of human nouns. The asterisk (*) indicates significant differences. Pearson’s Correlation between intentionality in each type of movement with the score for the use of human nouns (from 1 to 12) was higher in R movements (*r* = 0.57, *p* < 0.01), and lower in GD (*r* = 0.31, *p* = 0.004) and ToM movements (*r* = 0.24, *p* = 0.03).

An interaction effect between the fps and the type of movement was detected, *F*(2,162) = 8.68, *p* < 0.01, $\eta_{\text{p}}^{2}$ = 0.10 (see Figure 4, Panel A). Stickman figures with 10 fps (*M* = 3.52) obtained higher levels of attribution of intentionality than Stickman figures with 26 fps (*M* = 3.25) when they displayed ToM movements, *F*(1,81) = 3.91, *p* = 0.051; $\eta_{\text{p}}^{2}$ = 0.05. When the figures displayed GD movement, the Stickman figures with 10 fps (*M* = 2.40) obtained significantly higher levels of attribution of intentionality than the Stickman figures with 26 fps (*M* = 2.14), *F*(1,81) = 7.29, *p* = 0.008; $\eta_{\text{p}}^{2}$ = 0.08. A similar trend was observed in R movements, where figures with 10 fps (*M* = 1.48) were attributed higher levels of intentionality than figures with more fps (*M* = 0.54), *F*(1,81) = 18.11, *p* < 0.01; $\eta_{\text{p}}^{2}$ = 0.18.

Differences were also detected by type of movement *F*(2,162) = 321.94, *p* < 0.01, $\eta_{\text{p}}^{2}$ = 0.80, with the attribution of intentionality progressively decreasing from ToM movement (*M* = 3.39) to GD movement (*M* = 2.27), and from the latter to R movement (*M* = 1.01). Finally, a main effect of the number of fps was observed, with the group that observed Stickman figures with more fps attributing less intentionality (*M* = 1.98) than the group that observed Stickman figures with fewer fps (*M* = 2.47), *F*(1,81) = 17.36, *p* < 0.01, $\eta_{\text{p}}^{2}$ = 0.18.

From the written responses, it was possible to establish that 20 out of 45 (44.4%) participants who observed Stickman figures with more fps used human nouns. This value differed from 29 out of 38 (76.3%) who used human nouns when they observed the original Stickman figures with fewer fps, $\chi_{\left(1\right)}^{2}$ = 8.65, *p* < 0.01. As established in Experiment 1, those who watched Stickman figures tended to use more human nouns than would be randomly expected, *t*_(37)_ = 3.77, *p* < 0.01; however, part of the responses did not differ from what was randomly expected among those who watched the Stickman figures with more fps, *t*_(41)_ = 0.74, *p* = 0.46.

The use of nouns was statistically significant, *F*(1,80) = 21.73, *p* < 0.01, $\eta_{\text{p}}^{2}$ = 0.21 and its interaction with the type of movement was important, *F*(2,160) = 13.99, *p* < 0.01, $\eta_{\text{p}}^{2}$ = 0.15. When statistically controlling the effect of this variable, the results detected an interaction effect between the fps and the type of movement *F*(2,160) = 3.61, *p* < 0.05, $\eta_{\text{p}}^{2}$ = 0.04 (see Figure 4, Panel B). A *post hoc* analysis found that, in R movements, figures with less fps (*M* = 1.31) were attributed higher levels of intentionality than figures with more fps (*M* = 0.69), *F*(1,80) = 9.53, *p* < 0.01; $\eta_{\text{p}}^{2}$ = 0.11. When the figures displayed GD and ToM movements, the Stickman figures with less fps (*M* = 2.37) were not perceived to be different from the Stickman figures with more fps (*M* = 2.17), *F*(1,80) = 3.83, *p* = 0.054; $\eta_{\text{p}}^{2}$ = 0.05. Finally, when the figures displayed ToM movements, the Stickman figures with less fps (*M* = 3.48) had similar levels of intentionality than Stickman figures with higher fps (*M* = 3.29), *F*(1,80) = 1.91, *p* = 0.17.

The gradient of intentionality was maintained according to the type of movement, *F*(2,160) = 245.99, *p* < 0.01, $\eta_{\text{p}}^{2}$ = 0.76, with the attribution of intentionality being significantly higher in ToM movement (*M* = 3.38) than in GD movement (*M* = 2.27), *p* < 0.01; and the attribution of intentionality in the latter type of movement was greater than in R movement (*M* = 0.99), *p* < 0.01. Finally, a main effect of the number of fps was observed, with the group that watched Stickman figures with less fps attributing more intentionality (*M* = 2.38) than the group that observed Stickman figures with more fps (*M* = 2.05), *F*(1,80) = 9.29, *p* < 0.01, $\eta_{\text{p}}^{2}$ = 0.10.

### Discussion

In this experiment, we found that the fps interacts with the type of movement. Animations displayed at high fps were generally attributed less intentionality, but this did not occur in ToM movements. Animations with high fps also decreased the use of human nouns. When controlling the use of human nouns, the interaction effect was maintained, but there were only significant differences in R movement, where animations with more fps were attributed less intentionality. The same intentionality gradient found in our previous experiments was replicated.

Our results confirm those of Morewedge et al. (2007), who found that a higher apparent speed, given by animations with high fps rate, reduces the attribution of intentionality. However, we expected to observe this result in ToM movements. It is likely that the cue provided by the type of movement is so strong that it is not possible to diminish its effect on the attribution of intentionality beyond a certain level. As in this experiment we only used the Stickman figures, the attribution of intentionality in ToM is already lower than those found with the original Triangles. To further decrease the attribution of intentionality would require treating the ToM movement as GD, which would imply that the type of movement is not such a strong cue, something contrary to what we found in our experiments and in the literature.

It is interesting to note that animations with high fps in R movements were attributed a level of intentionality similar to that of the Triangles used in Experiment 1. This possibly suggests that high apparent speed is a strong cue that counteracts the effect of the type of figure in R movements.

## General Discussion

Analyzing the findings, we can state that results reported by previous research are confirmed because in our three experiments we observed the same gradient of intentionality among types of movement: ToM > GD > R (Castelli et al., 2000; Salter et al., 2008; Klein et al., 2009; White et al., 2011; Zwickel et al., 2011). However, the critical aspects of our results were the subtle interactions between the variables studied and the types of movements.

In Experiment 1, taking into account prior studies in which no differences in terms of mentalistic explanations were found between human and non-human silhouettes displaying GD movements (Michotte, 1950; Rimè et al., 1985), we expected to find no differences in attribution of intentionality between Triangles and Stickman deploying the same type of movement. However, we did expect higher levels of attribution of intentionality in the group that watched Stickman figures in ToM and R movements, because an anthropomorphized figure could favor the intentional stance under these types of movements (Krach et al., 2008; Thellman et al., 2017). The results partially confirmed our hypotheses. The group that observed Stickman figures deploying R movement attributed greater intentionality scores. As expected, no differences were found in GD movements, but, contrary to our expectations, in ToM movements, the group that watched Stickman figures gave lower intentionality scores than the group that watched Triangles. We conjecture that this result can be explained by the design of the figure or the discrepancy between the figure and the movements it performs rather than by the anthropomorphism of the figure. Both types of figures in our experiment have a simple design, which perhaps reduced the attribution of intentionality. Working on how humans ascribe humans qualities to artificial devices; such as robots; Hegel (2012) found that people consider that robots with a more sophisticated design have more social capabilities, such as honesty, intelligence, and emotions. In our experiment, the figures had a simple design, so they could have an effect similar to robots with an unsophisticated design. This would make these figures less likely to receive intentional descriptions. However, it is not clear why the attribution of intentionality to the Stickman design decreases compared to the Triangle design when they performed ToM movements, since both have a simple design. We presume that a more adequate explanation is the discrepancy between the static and smiling faces of our Stickman figures and the movements they are performing. A poorly designed face can send unintended or inaccurate messages (Donath, 2001) that reduce attentional resources. It is possible that the face of the figures distracted the participants from the main cue (the type of movement) during the attribution of intentionality. This negative effect probably occurred in ToM movements because these movements are more complex and more difficult to interpret than R and GD movements, which do not need elevated attentional resources or additional analyses.

In Experiment 2, we manipulated the way the figures were labeled (Triangles referred to as Mister X. and Mister Y.). Considering that labels can generate greater emotional attribution (Oatley and Yuill, 1985) and that the instructions given to participants can favor an intentional stance (Wiese et al., 2013; Özdem et al., 2017), we expected that the group with labels that humanize the figures would assign higher intentionality scores in all three types of movements. The results partially confirmed our hypothesis. The intentionality scores were higher in GD and R movements in the group where the figures were referred to as people. However, in ToM movements, the labeling of the figures did not affect intentionality scores. This finding again demonstrates the subtle interaction between the type of movement with a verbal cue present in the instruction. Although the label increased the attribution of intentionality in R and GD movements, figures that showed ToM movement were not affected by the label present in the instructions. We can assume that the attribution of intentionality system operates with a hierarchy in which the type of movement is the most relevant aspect, while the label can only affect the attribution of intentionality in R and GD movements.

Finally, in Experiment 3 we manipulated the apparent speed of the figures (Stickman). Considering that humans perceive that objects or agents are more likely to possess mental states when they move at speeds similar to natural human movement, compared to when they move at faster speeds (Morewedge et al., 2007), we expected that a higher apparent speed would result in low intentionality scores in all three types of movement. Our hypothesis was, again, partially correct. We found that participants attributed less intentionality to high fps (higher apparent speed) animations in R and GD movements. However, this was not the case in ToM movements, in which no differences were found between high and low fps conditions. One way to explain this result is that intentionality was negatively affected by the use of Stickman figures from Experiment 1. We hypothesized that anthropomorphized figures tend to limit the attributed intentionality when they perform ToM movements and that, even though the type of movement is a strong signal to attribute intentionality, it can be diminished by increased apparent speed (perceptual cue) only when the movement is R or GD. It is also interesting to note that the high fps animations in the R movements were attributed a similar intentionality score than that of the group that observed the Triangles in Experiment 1. This suggests that higher apparent speed may counteract the effect of the human-shaped figure in R movements.

Our experimental manipulations also affected the use of human nouns. The Stickman figures in Experiment 1 and the Triangles referred to as people in Experiment 2 increased the use of human nouns in participants’ responses, while in Experiment 3, the Stickman with high fps reduced the use of human nouns compared to the Stickman with lower fps. We found that the use of human nouns was a variable that covariates with intentionality scores. When we statistically controlled the use of human nouns in Experiment 1, the difference in intentionality scores in the R movements disappeared, while the difference between groups increased in the ToM movements. This suggests that human nouns generated by the Stickman favored the attribution of intentionality, even in ToM movements, but, as previously discussed, the possible discrepancy between the figure face in relation to the complex movements of ToM animations could also have negatively affected intentionality scores. In Experiment 2, when the use of human nouns was controlled, the interaction between the type of movement and the figure label disappeared. However, the group that watched the figures labeled as people still had higher intentionality scores in the R movements, but not in the GD movements. This indicates that the effect of figure labeling is still present to some degree, even though the use of human nouns is neutralized in their responses. In Experiment 3, the differences in GD disappeared when the use of nouns was controlled, while in the R movements, the group that watched the animations with more fps still attributed lower intentionality scores. We again observe that by controlling the use of nouns, the differences between the groups are reduced, so we conclude that favoring the use of human nouns in participants’ responses increases the attribution of intentionality, although not significantly in the ToM movements. Further analysis found that in ToM movements the intentionality score and the use of nouns did not correlate to each other. In contrast, in R movements, the correlation between the intentionality score and the use of human nouns was strong in Experiment 1, and moderate in Experiments 2 and 3, while their correlation in the GD movements was weak.

This difficulty in increasing the attribution of intentionality in ToM movements may be because these types of movements have more perceptual information. When perceptual information is low or ambiguous, people use their previous expectations in the attribution of intentionality (Chambon et al., 2011, 2017; Koul et al., 2019) or resort to conceptual cues instead of perceptual cues (Gelman et al., 1995). We can consider that R and GD movements have less perceptual information compared to ToM movements, so participants resort to other cues present in the animations (Shape of the figure or label of the figure) and the previous expectations that these cues generate. It is possible that participants will use these cues to a lesser extent in ToM movements due to the high perceptual information they possess. This could also explain why participants attribute more intentionality than expected in R movements, since they would resort to prior expectations generated by the shape of the figure and the label. It would be incongruent if something with a name or a human shape were to engage in unintended behavior.

The results lead us to assume, in the context of this or similar tasks, the existence of a hierarchical system of detectors that are activated to identify whether or not the object or figure acts in an intentional way, and at which level of complexity it does so. This set of detectors is sensitive to the type of movement. Once the type of movement is detected, other detectors of perceptual (shapes and apparent speed) and semantic (labels) cues are activated, which act differently depending on the type of movement. In our case, R movements are movements performed mainly by physical objects, which follow rules of cause and effect. Any manipulation that brings them closer to humans (the form, the way they are referred to, or the speed of movement) will increase the intentionality attributed to them. In the case of ToM and GD movements, two types of movements that are usually classified as intended behavior, people should be less permeable to the effect of these semantic and perceptual cues. However, in circumstances where a strange perceptual cue appears, such as a rigid face inconsistent with movement, instead of maintaining intentionality, the detector would decrease it.

It is worth noting that our experiments used a standard procedure for giving the instructions, and no examples or explanations were provided that encouraged (or discouraged) participants to use certain types of descriptions. In that regard, we reduced the potential variability that we suspected could have been a source of variability. Average intentionality scores in other studies with control groups consisting of subjects with typical neurodevelopment (Castelli et al., 2000; Salter et al., 2008; Klein et al., 2009; White et al., 2011; Zwickel et al., 2011), have varied between 3.8 and 2.73 in ToM movement; between 2.64 and 2.21 in GD movement; and between 0.47 and 0.12 in R movement. Our results maintained the same gradient previously observed in intentionality scores. However, in R movement, the use of humanized figures such as Stickman figures or Triangles referred to as people increased the average values of intentionality (between 1.48 and 1.20 without controlling the covariate; and between 1.31 and 0.69 when the covariate was controlled, respectively). The increment in scores could be attributed to the incorporation of perceptual or semantic elements that humanized the objects. As for the ToM movement of Triangles referred to as figures and Triangles referred to as people, intentionality averages were higher than those previously reported (between 3.89 and 4.08 without controlling the covariate; and between 3.98 and 3.99 when the covariate was controlled, respectively). Thus, the abstract figures displaying ToM movement used in our experiments would have high levels of intentionality, regardless of how they were referred to. Finally, regarding the GD movement, we did not observe any substantial changes in intentionality scores, because the values were distributed within the previously studied ranges.

This study has two main implications. The first one is that some variables may affect the results in the Frith-Happé triangles task and other similar tasks (e.g., Ramsey and Hamilton, 2010; Surian and Geraci, 2012; van Buren et al., 2015). The second one is that attribution of intentionality is not always straightforward, since including more conceptual or perceptual cues, as well as increasing their effect, will not always have a positive result in the attribution of intentionality. For example, in the uncanny valley effect (Kätsyri et al., 2015), enhancing the human resemblance of a stimulus can make it more appealing, but, when the human resemblance is further increased, an unpleasant sensation can be produced when the stimulus is observed. Along the same line, certain cues might have unexpected effects even when they are irrelevant to the task. One example is the wolfpack effect (Gao et al., 2010; van Buren et al., 2015), in which whether the elements in the background are pointing toward the agent, causes subjects to perceive that the agent is being chased, even when the subjects know that these elements are irrelevant to the task. In our study, the main cue is the type of movement, and although it is assumed that other cues do not provide relevant information to develop the task, they still influence the attribution of intentionality. Another example in which the attribution of intentionality varies unexpectedly can be observed in the Knobe effect (Knobe, 2003), which consists in an asymmetry in which people are much more prone to blame an agent for negative side effects than to praise this agent for positive side effects. When an agent’s actions produce negative side effects, people are more likely to say that the effect was intentional and to blame the agent for the consequences; while the action that generates a positive side effect is perceived as having less intentionality and, therefore, being less praiseworthy.

These findings may be limited by our modification of the original figures. Although we affected the attribution of intentionality with the Stickman figures, the result was contrary to our hypothesis and the studies reviewed. The reason for this may be that some parts of the figure features (eyes, smile, legs, and arms) did not match or were not credible with the actions they performed. We believe that the static face present in Stickman figures may be the trigger that induced the observer to attribute less intentionality. Thus, our suggestion is to try to use Stickman figures without a face or to make the faces have facial expressions in accordance with the actions performed by the figures. We also suggest using the same faces of the Stickman figures in the Triangles. Taking into account our results, we hypothesize that the Triangles should show lower levels of intentionality in this situation.

Another aspect not studied here is the additive effect of the shape, label and apparent speed in the attribution of intentionality in R movements. Regardless of the use of human nouns, experimental manipulation had a stable effect. Participants perceived more intentionality with anthropomorphized figures when they were labeled as human, and when they moved within the ranges of what is considered human speed (low fps). The possibility of studying how the three factors (shape, label, and apparent velocity) are simultaneously integrated into an experiment could affect the levels of intentionality more significantly compared to conditions where two of them are integrated.

We recommend interpreting these results within the context of the task used in this experiment, that is, the attribution of intentionality to the movement of simple figures, and their interaction with other variables in a two-dimensional context.

## Conclusion

This study aimed to explore whether some conceptual and perceptual cues influence the attribution of intentionality when the type of movement is the main and most important cue. In all three experiments, the attribution of intentionality to non-human moving objects was researched. Following Abell et al. (2000), three movement conditions (R, GD, and ToM) were used. Experiment 1 explored the attribution of intentionality to Stickman figures in comparison to geometric figures, revealing that in the ToM condition participants attributed more intentionality to geometric figures than to Stickman figures. However, in R movements, the Stickman figures obtained higher intentionality scores. Experiment 2 showed that when the figures were presented with human name labels, the attribution of intentionality in the conditions of R and GD movements increased, but not in ToM movements. In Experiment 3, the figures move at a higher (unnatural) speed than in Experiment 1. This condition decreased the attribution of intentionality in R and GD movements but not in the ToM movements. In addition, Stickman figures and labels promote the use of human nouns in participants’ responses, while a high apparent speed reduces their use.

The use of human nouns was associated to intentionality scores significantly in R movements, but at a lesser extent in GD and ToM movements. When the effect of human nouns was controlled in the analyses, the Stickman figures only reduced the intentionality scores in ToM movements, the label only increased the scores in R movements, and the animations with high apparent speed only reduced the scores in R movements.

We conclude that, although the type of movement is the most important cue in this sort of task, the tendency to attribute intentionality to figures is affected by the interaction between perceptual and semantic cues (figure shape, label, and apparent speed).

## Data Availability Statement

The data used to support the findings of this study are available from the corresponding author upon request.

## Ethics Statement

The studies involving human participants were reviewed and approved by Comité de Ética Científica de la Universidad de Talca. The patients/participants provided their written informed consent to participate in this study.

## Author Contributions

All authors contributed to the elaboration and design of the experiments, the data acquisition, the analysis and interpretation of the results. All authors have been involved in drafting the manuscript and revising it critically and have given final approval of the version to be submitted to review.

## Conflict of Interest

The authors declare that the research was conducted in the absence of any commercial or financial relationships that could be construed as a potential conflict of interest.

## Supplementary Material

DATA SHEET S1 Intentionality scores and the use of human nouns in the three experiments.
